# Endometrium evaluation with high-field (3-Tesla) magnetic resonance imaging in patients submitted to uterine leiomyoma embolization

**DOI:** 10.1590/S1679-45082013000100011

**Published:** 2013

**Authors:** Monica Amadio Piazza Jacobs, Felipe Nasser, Eduardo Zlotnik, Marcos de Lorenzo Messina, Ronaldo Hueb Baroni

**Affiliations:** 1 Post-graduation Program in Abdominal Imaging, Hospital Israelita Albert Einstein, São Paulo, SP, Brazil; 2 Intervention Radiology Department, Hospital Israelita Albert Einstein, São Paulo, SP, Brazil; 3 Gynecology and Obstetrics Department, Hospital Israelita Albert Einstein, São Paulo, SP, Brazil; 4 Magnetic Resonance Unit, Imaging Department, Hospital Israelita Albert Einstein, São Paulo, SP, Brazil

**Keywords:** Uterine artery embolization, Fertility, Leiomyoma, Magnetic resonance imaging

## Abstract

**Objective:**

To evaluate the endometrial alterations related to embolization of uterine arteries for the treatment of symptomatic uterine leiomyomatosis (pelvic pain and/or uterine bleeding) by means of high-field (3-Tesla) magnetic resonance.

**Methods:**

This is a longitudinal and prospective study that included 94 patients with a clinical and imaging diagnosis of symptomatic uterine leiomyomatosis, all of them treated by embolization of the uterine arteries. The patients were submitted to evaluations by high-field magnetic resonance of the pelvis before and 6 months after the procedure. Specific evaluations were made of the endometrium on the T2-weighted sequences, and on the T1-weighted sequences before and after the intravenous dynamic infusion of the paramagnetic contrast. In face of these measures, statistical analyses were performed using Student's *t* test for comparison of the results obtained before and after the procedure.

**Results:**

An average increase of 20.9% was noted in the endometrial signal on T2-weighted images obtained after the uterine artery embolization procedure when compared to the pre-procedure evaluation (p=0.0004). In the images obtained with the intravenous infusion of paramagnetic contrast, an average increase of 18.7% was noted in the post-embolization intensity of the endometrial signal, compared to the pre-embolization measure (p<0.035).

**Conclusion:**

After embolization of the uterine arteries, there was a significant increase of the endometrial signal on the T2-weighted images and on the post-contrast images, inferring possible edema and increased endometrial flow. Future studies are needed to assess the clinical impact of these findings.

## RESUMO

**Objetivo:**

Avaliar alterações endometriais relacionadas à embolização de artérias uterinas para tratamento da leiomiomatose uterina sintomática (dor pélvica e/ou sangramento uterino), por meio de ressonância magnética de alto campo (3 Tesla).

**Métodos:**

Estudo longitudinal e prospectivo, no qual foram incluídas 94 pacientes com diagnóstico clínico e por imagem de leiomiomatose uterina sintomática, todas tratadas por meio de procedimento de embolização das artérias uterinas. As pacientes foram submetidas a avaliações por ressonância magnética de alto campo da pelve antes e 6 meses após o procedimento. Foram realizadas avaliações específicas do endométrio nas sequências ponderadas em T2 e nas sequências ponderadas em T1, antes e após a infusão endovenosa dinâmica do meio de contraste paramagnético. Diante dos resultados dessas medidas, foram realizadas análises estatísticas por meio de teste *t* de Student para comparação dos resultados obtidos antes e após o procedimento.

**Resultados:**

Observou-se um aumento médio de 20,9% no sinal endometrial nas imagens ponderadas em T2 obtidas após o procedimento de embolização das artérias uterinas, quando comparadas com a avaliação pré-procedimento (p=0,0004). Nas imagens obtidas com a infusão endovenosa do meio de contraste paramagnético, foi observado um aumento médio de 18,7% na intensidade de sinal endometrial pós-embolização de artérias uterinas, quando comparadas com a medida pré-embolização (p<0,035).

**Conclusão:**

Após a embolização de artérias uterinas, houve significativo aumento do sinal endometrial nas imagens ponderadas em T2 e nas imagens pós-contraste, inferindo possível edema e aumento do fluxo endometrial. Estudos futuros são necessários para avaliar o impacto clínico destes achados.

## INTRODUCTION

Leiomyomas are the most frequent benign tumors of the female genital tract, varying from 20 to 40% among women in their reproductive age^(1,2)^. Currently, there are a few options for treatment of leiomyomatosis besides surgery, such as pharmacological medical therapy and radiological interventions such as uterine artery embolization (UAE).

UAE is considered an important therapeutic alternative for symptomatic leiomyomas (pelvic pain and/or uterine bleeding). Ideal candidates for this procedure are symptomatic patients that desire to preserve the uterus, or those who cannot or do not wish to be submitted to surgical treatment^(3)^.

Leiomyomas are almost exclusively nourished by the uterine arteries. The objective of the procedure is to carry particulate material in both uterine arteries, in order to provoke ischemic alterations in the myomas, while avoiding uterine lesions^(4–9)^.

Although studies have evaluated the relationship between uterine leiomyomas and fertility, the fundamental mechanism by which uterine leiomyomatosis affects female reproductivity remains undetermined. Some hypotheses suggest that leiomyomas determine dysfunctional uterine contractility, which can interfere in the migration of the sperm, in transportation of the ovule, or in nidation^(10)^. Leiomyomas may also be related to failures of implantation or miscarriage due to focal vascular endometrial disorders, endometrial inflammation, and secretion of vasoactive substances^(10–12)^.

Today, studies on the effects of UAE in the ovaries and endometrium, and on possible alterations in fertility and subsequent pregnancy, are still limited^(3)^.

Magnetic resonance (MRI) is considered an excellent imaging method for evaluating myomas, due to its noninvasive characteristics, absence of ionizing radiation, and high contrast resolution for the evaluation of pelvic organs^(13)^. The method is also used for pre- and post-embolization evaluation of uterine myomas^(14)^. High-field MRI (3-Tesla, with double the magnetic field strengh of the most commonly used magnets) provides better general quality of images, since it generates a higher signal (in intensity of the brightness) and therefore, allows better definition of small structures.

## OBJECTIVE

To evaluate endometrial alterations that occurred after the UAE therapeutic procedure by means of high-field (3-Tesla) MRI.

## METHODS

This was a longitudinal and prospective study carried out over a period of 2 years at our service, approved by the Institutional Ethics Committee, #08/926.

All the patients read and signed the Informed Consent Form.

Inclusion criteria were women of reproductive age, with a desire to reproduce, with a clinical and radiological diagnosis of symptomatic uterine leiomyomas (presence of uterine bleeding and/or pelvic pain), candidates for UAE treatment, and with a high-field MRI evaluation performed after the therapeutic procedure.

Exclusion criteria were patients who had not had a high-field MRI evaluation before or after the procedure, those with high-field MRI tests bearing technical artifacts hindering appropriate analysis of the images, or with inadequate endometrial thickness for correct evaluation.

Of an initial sample with 130 patients, 36 were excluded. The final case series was made up of 94 patients, whose most relevant clinical and demographic data are listed on table 1.

**Table 1:** Demographic and clinical data of patients submitted to embolization of uterine arteries

Patient's data	Range (mean)
Age	27-48 (37.2)
Parity	0-4 (0.4)
Number of myomas	1-9 (4.1)

Patients were submitted to the UAE procedure as per the institutional protocol previously described in detail in other publications^(15,16)^.

Imaging tests were performed on high-field MRI device (Siemens Magneton Trio, Erlangen, Germany) in two instances: one week before the (UAE) procedure and approximately 6 months after. All the women were instructed to schedule the high-field MRI evaluation between the 10^th^ and 20^th^ day of their menstrual cycle.

The protocol used was the same in both evaluations: T2-weighted fast spin echo images on axial, coronal, and sagittal planes (4-mm thickness, 1-mm interval, TR/TE=3.600/159ms); T1-weighted axial sequences in-phase and out-of-phase (4-mm thickness, 1-mm interval, TR/TE=155/2.43ms and 155/1.26ms); and T1-weighted sagittal sequences before and after the gadolinium-based intravenous paramagnetic contrast infusion (gadopentetate dimeglumine, Magnevistan^®^, Bayer), with a 0.1 mmol/kg dose, and image acquisition times of 0, 30, 60, 90, and 120 seconds after the end of the contrast infusion.

**Figure 1:**
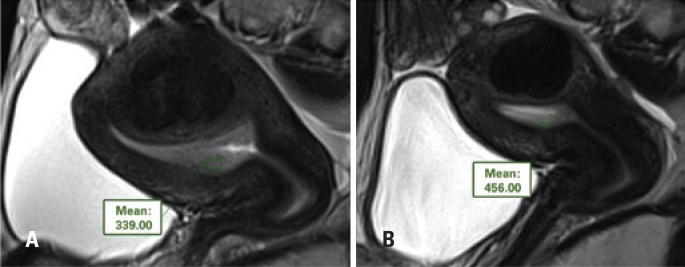
T2-weighted sagittal images demonstrating evaluation of the endometrial signal by ROIs before (A) and after (B) embolization of uterine arteries, with increased signal in (B)

**Figure 2:**
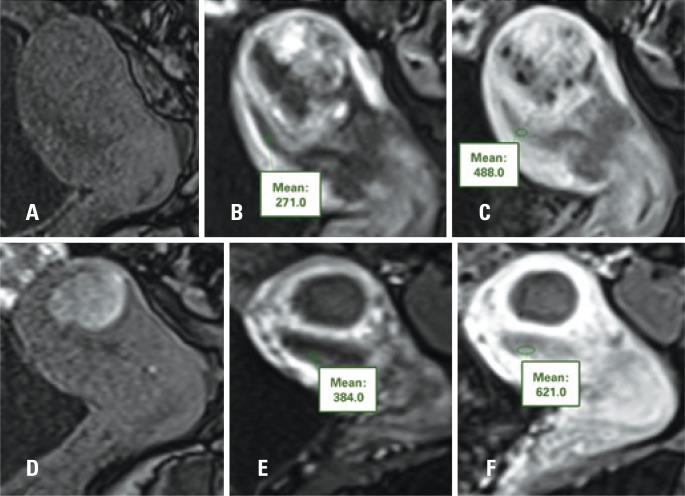
(A, B and C) Measurements of the endometrial signal by ROIs before embolization of uterine arteries. (A) Time 0, (B) 30 seconds and (C) 120 seconds after intravenous infusion of paramagnetic contrast medium. (D, E and F) Measurements of the endometrial signal by ROIs, after UAE. (A) Time 0, (B) 30 seconds and (C) 120 seconds after intravenous infusion of paramagnetic contrast medium. Observe increased endometrial signal intensity when compared to pre-UAE evaluation

The images were evaluated and interpreted by a radiologist experienced in abdominal imaging. The methods used for endometrial evaluation were the measurement of the endometrial signal, performed by means of the use of the region of interest (ROI) on T2-weighted sagittal images (to evaluate the degree of endometrial hydration, since the greater the hydric content, the greater the T2 signal), and dynamic sagittal images before and after intravenous infusion of the paramagnetic contrast medium (to evaluate the degree of endometrial blood perfusion). At least 40% of the endometrial thickness was included in the measurement, avoiding the inclusion of parts of the myometrium or of the uterine cavity. A second radiologist, specialized in abdominal imaging, confirmed the correct position of the ROIs (Figures 1 and 2).

Once the measurements were made, calculations were performed of the mean, standard deviation, minimum and maximum values of each comparable step, that is, T2-weighted sequences pre- and post- UAE, and all five dynamic phases after the contrast medium intravenous infusion, also before and after the UAE procedure. Student's *t* test was done to compare these measurements in order to evaluate the presence or not of significant differences in the endometrial signal intensity (significance level of 0.05).

## RESULTS

In the MRI evaluations, a statistically significant difference was observed in the endometrial signal intensity on T2-weighted images, as well as in the post-contrast endometrial mean enhancement intensity, when comparing pre- and post-procedure tests.

**Figure 3:**
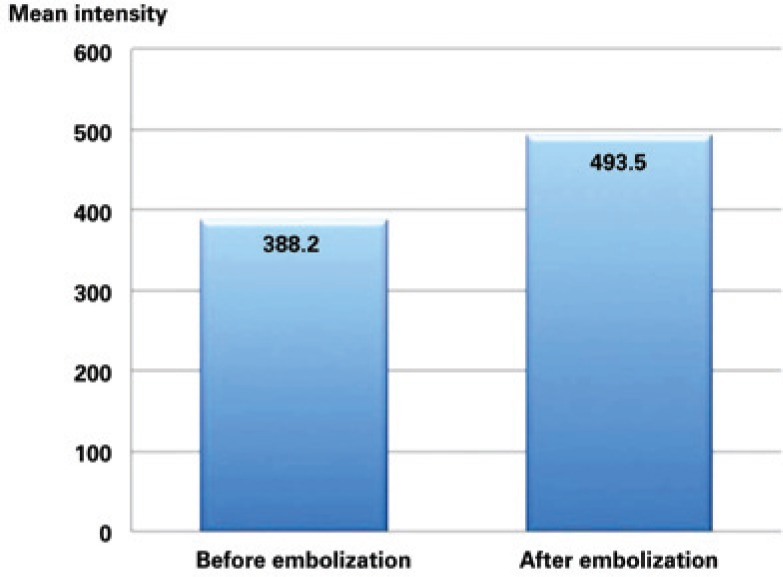
Mean value of endometrial signal intensity on T2-weighted magnetic resonance images. Note the significant increase of endometrial signal intensity (inferring edema) after embolization of arteries uterines (p=0.0004)

In the pre-embolization tests, the mean value of endometrial signal intensity on the T2-weighted sequences was 388, with a standard deviation of 121, minimum value of 159 and maximum of 701. In post-embolization tests, the mean value of endometrial signal intensity on the T2-weighted sequences was 493, with a standard deviation of 244, minimum value of 59 and maximum of 1.396 (mean increase of 20.9%; p=0.0004) (Figure 3).

**Figure 4:**
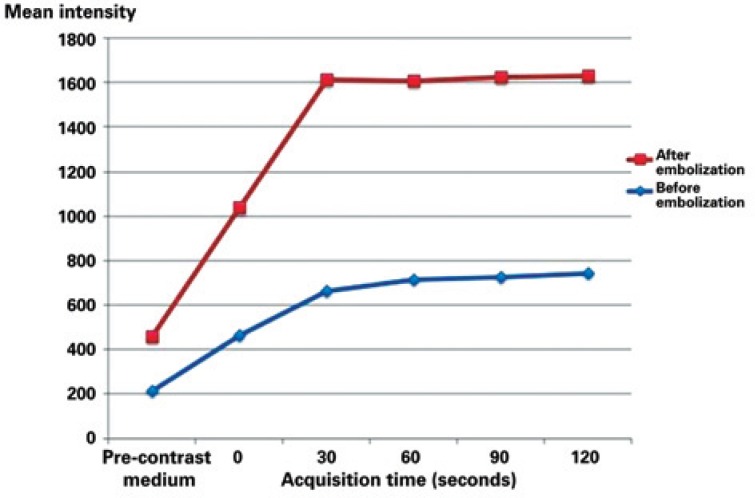
Mean values of endometrial signal intensity before and after infusion of paramagnetic contrast medium. Note the significant mean increase of endometrial signal intensity (inferring more enhancement/vascularization) after arterial embolization (p<0.035)

Analyzing the images of the pre- and post-intravenous contrast medium infusion in the pre-UAE, the mean value of the endometrial signal intensity was 215 in the pre-contrast series, 464 at the 0 timepoint, 661 at 30 seconds, 713 at 60 seconds, 725 at 90 seconds, and 740 at 120 seconds. The same values in the post-UAE evaluation were 246 in the pre-contrast series, 572 at the 0 timepoint, 953 at 30 seconds, 892 at 60 seconds, 898 at 90 seconds, and 888 at 120 seconds (mean increase of 18.7%; p<0.035) (Figure 4).

Summarizing, the endometrial intensity signal values were higher in the images evaluated after the UAE procedure, both on the T2-weighted images and on the images obtained after the dynamic infusion of paramagnetic contrast.

## DISCUSSION

The current study evidenced that after UAE there is a significant increase of the T2 signal (inferring a greater degree of hydration or tissue inflamation) and higher post-contrast enhancement (inferring greater blood inflow) in the endometrium.

After the UAE, a series of inflammatory processes is triggered, resulting in ischemia and necrosis of the leiomyomas^(17)^. This necrosis process often involves the release of cytokines, especially the tumor necrosis factor (TNF). TNF-α is a cytokine synthesized and released by a series of cells, especially macrophages. Most organs of the human body are affected by this cytokine, which represents inflammation, among other functions^(17,18)^. Tracey and Cerami^(19)^ suggested two beneficial functions related to TNF-α: low levels of this cytokine may help maintain homeostasis and promote renovation and substitution of tissues that suffered lesion or senescence, and since they are acute phase proteins, may also increase vascular permeability^(17–19)^.

Different types of endometrial cells, including epithelial cells, express receptors for TNF-α, increasing the biosynthesis of local endometrial estrogen and transforming estrogen into a more active metabolite. These effects may have an impact on many physiological and pathological processes that occur within the endometrium^(17–19)^.

Within this context, it is pertinent to consider that after UAE, inflammatory and hormonal alterations are triggered that ultimately would increase endometrial vascular permeability, with a good correlation of these findings with the results of our study, in which one can infer the occurrence of endometrial edema (increase signal on the T2-weighted images) and greater blood flow (increased signal on the images after the intravenous infusion of the contrast medium) after the UAE.

There is great controversy in literature as to the impact on the fertility of patients submitted to UAE^(4–6)^. Although this aspect has not been the objective of this study, the results found here may open a new front in studies on endometrial alterations related to UAE. It is possible (but not proven) that a more vascularized endometrium may have a greater chance of enabling a healthy gestation^(4–6)^.

Some limitations of the current study should be pointed out. Although the evaluation of the images was made anonymously and randomly, the presence of necrosis in myomas may be easily identified by the radiologist, generating a possible bias of analysis by the suspicion of which phase of the test was in question (preor post-embolization). However, we believe that this fact did not interfere in the results, since the measurements and positions of the ROIs were checked twice.

Additionally, we did not correlate the image findings with the results of endometrial biopsies. New studies with clinical/histological correlations should be performed to confirm these results.

## CONCLUSION

This study showed that high-field MRI is capable of detecting significant endometrial alterations after UAE, with an increase of the endometrial signal on T2-weighted images and after the injection of paramagnetic contrast media, inferring, respectively, edema and increased blood flow of the endometrium.
